# Prevalence of domestic violence and associated factors among pregnant women using the Brazilian Unified Health System: a cross-sectional study, Curitiba, 2018-2020

**DOI:** 10.1590/S2237-96222026v35e20250920.en

**Published:** 2026-06-07

**Authors:** Julia Valoski, Ádelin Olívia Lopes Joly Rodrigues, Isabela Cristina Santos Freire de Paula, Renata Iani Wernek, Juliana Schaia Rocha Orsi

**Affiliations:** ¹ Pontifícia Universidade Católica do Paraná, Curitiba, PR, Brazil Pontifícia Universidade Católica do Paraná Curitiba PR Brazil

**Keywords:** Domestic Violence, Pregnant People, Gender-Based Violence, Health Services, Cross-Sectional Studies, Violencia Doméstica, Personas Embarazadas, Violencia de Género, Servicios de Salud, Estudios Transversales

## Abstract

**Objective:**

To analyze prevalence and factors associated with domestic violence among pregnant women using the public health network of Curitiba, based on data from the Curitiba Maternal and Child Health Cohort project.

**Methods:**

This was a cross-sectional observational study, conducted with pregnant women using the Brazilian Unified Health System in Curitiba between 2018 and 2020. Demographic, socioeconomic, emotional and obstetric variables were analyzed. The dependent variable was having experienced domestic violence (physical, psychological or sexual). Absolute and relative frequencies were calculated, and the chi-square test with Bonferroni correction was applied to select variables with a p-value<0.20. Independent associations were estimated using Poisson regression with robust variance, presenting prevalence ratios (PR) and 95% confidence intervals (95%CI).

**Results:**

194 pregnant women were analyzed. Prevalence of domestic violence was 27.5%, with 73.1% being physical and 75.0% psychological. Violence was associated with feelings of sadness (PR 2.83; 95%CI 1.46; 5.48; p-value 0.002), household arrangement (prevalence of violence was lower among pregnant women living with partners: PR 0.51; 95%CI 0.29; 0.92; p-value 0.025), and with intermediate gestational risk (PR 2.73; 95%CI 1.54; 4.84; p-value 0.011).

**Conclusion:**

Domestic violence was frequent among pregnant women using the public health network in Curitiba and was associated with emotional vulnerability, living without a partner, and intermediate gestational risk. These findings reinforce the need for intersectoral actions and psychological and social support during prenatal care in order to identify and manage violence.

## Resumen

**Objetivo:**

Analizar la prevalencia y los factores asociados a la violencia doméstica en embarazadas atendidas en la red pública de salud de Curitiba, con base en datos del proyecto Cohorte de Salud Maternoinfantil de Curitiba.

**Métodos:**

Estudio observacional transversal, realizado con embarazadas atendidas en el Sistema Único de Salud en Curitiba entre 2018 y 2020. Se analizaron variables demográficas, socioeconómicas, emocionales y obstétricas. La variable dependiente fue la experiencia de violencia doméstica (física, psicológica o sexual). Se calcularon frecuencias absolutas y relativas, y se aplicó la prueba de chi-cuadrado con corrección de Bonferroni para seleccionar variables con un valor p<0,20. Las asociaciones independientes se estimaron mediante regresión de Poisson con varianza robusta, presentando razones de prevalencia (RP) e intervalos de confianza del 95% (IC95%).

**Resultados:**

Se analizaron 194 mujeres embarazadas. La prevalencia de violencia doméstica fue de 27.5%, siendo 73.1% física y 75.0% psicológica. La violencia se asoció con sentimientos de tristeza (RP 2.83; IC95% 1.46; 5.48; valor p 0.002), arreglo del hogar (las mujeres embarazadas que vivían con su pareja tuvieron prevalencia menor de violencia: RP 0.51; IC95% 0.29; 0.92; valor p 0.025), y riesgo gestacional intermedio (RP 2.73; IC95% 1.54; 4.84; valor p 0.011).

**Conclusión:**

La violencia doméstica fue frecuente entre las mujeres embarazadas atendidas en la red pública de salud de Curitiba y se asoció con vulnerabilidad emocional, vivir sin pareja y riesgo gestacional intermedio. Estos hallazgos refuerzan la necesidad de acciones intersectoriales y apoyo psicológico y social durante la atención prenatal para la identificación y manejo de la violencia.

**Ethical aspects**

This research respected ethical principles, having obtained the following approval data:

Comitê de ética em pesquisa: Pontifícia Universidade Católica do Paraná. Número do parecer: 6.498.474. Data de aprovação: 9/11/2023. Certificado de apresentação de apreciação ética: 90052118.9.3001.0101.

Comitê de ética em pesquisa: Secretaria Municipal de Saúde de Curitiba. Número do parecer: 6.588.994. Data de aprovação: 18/12/2023. Certificado de apresentação de apreciação ética: 90052118.9.0000.0020.

## Introduction 

Violence against women is a historical and cultural issue that is endemic throughout the world and can manifest itself in various ways such as verbal and psychological abuse, physical aggression, torture and death ^1^^,^^2^. This form of violence occurs due to gender inequalities. These are built based not only on biological differences, but above all on the socially assigned role of men in a civilization shaped by a masculinized and patriarchal ideal, whereby men are seen as central figures while women play a secondary role ^2^. With various changes over time, such as technological and sociocultural development, as well as human evolution itself, many struggles, movements and discussions have taken place regarding patriarchy ^1^^-^^3^.

The increase in reports of domestic violence, sexual violence and other forms of violence against women highlights the persistence of this problem as a major public health challenge. In 2023, 302,856 reports of this type of violence were recorded in Brazil, representing a significant increase compared to the 216,024 cases recorded in 2022. In addition to being a sociocultural problem, domestic violence also falls under the umbrella of public health issues ^4^^,^^5^.

In Brazil, despite the progress made since the enactment of Law No. 11340 (Maria da Penha Law) in 2006, as a State response to these issues ^6^, violence against women remains rooted in sociocultural patterns that limit change. Furthermore, the effectiveness of institutional responses depends not only on punishing the guilty, but also on expanding prevention, early identification and support strategies ^7^^,^^8^.

With regard to pregnant women, violence takes on even greater relevance, as it contributes to increased maternal morbidity and mortality rates. In this sense, the systematic incorporation of identifying violence against pregnant women into health actions can contribute to the visibility of this issue and enable more effective interventions to reduce these rates ^9^^,^^10^.

Basic health services work in detecting, preventing and acting against violence. These services are considered the gateway to addressing this issue and establishing linkage with women during prenatal care, identifying the situation and encouraging reporting ^7^^,^^11^. In Brazil, the scarcity of studies on the occurrence and types of violence against women during pregnancy may be related to the difficulty women encounter in reporting violence, in both institutional and research contexts, which may contribute to underreporting of this phenomenon ^7^^,^^12^.

This study aimed to analyze prevalence of domestic violence and associated factors among pregnant women receiving care in the Brazilian Unified Health System in Curitiba.

## Methods 

Design 

This is a cross-sectional observational study, based on a sample from the Curitiba Maternal and Child Health Cohort. We only used information collected during the gestational period, enabling cross-sectional analysis, selected to estimate prevalence of domestic violence and identify factors associated with pregnancy ^13^.

Setting

The Curitiba Maternal and Child Health Cohort is an observational and longitudinal mixed cohort study (historical and concurrent) focused on pregnant women and their children, all linked to the public health network of Curitiba, Paraná, Brazil. The detailed methodology of the cohort, including the data collection instrument and its validation process, is described in its methodological protocol ^11^. The concurrent cohort involves the recruitment of pregnant women, at any stage of pregnancy, aged 16 years or older, residing in Curitiba.

The objective of the Curitiba Maternal and Child Health Cohort is to explore various aspects of the health of pregnant women and their children during the first 1,000 days of life. This contributes to understanding factors that impact child development and maternal well-being within the context of the Curitiba Mother Network (*rede Mãe Curitibana*).

Participants 

This research was based on a convenience sample, composed of all pregnant women participating in the Curitiba Maternal and Child Health Cohort study between August 2018 and May 2020 (n=244) who answered the question about domestic violence (n=194). No sample size calculation was performed, since this study is a secondary analysis of available data.

Participants who agreed to take part in the study signed an informed consent form. Data collection was carried out in person by a previously trained team, using a questionnaire structured in sections of variables. The complete protocol can be consulted for more details about the study ^13^.

Of the total 244 eligible pregnant women, 50 (20.49%) did not answer the question about domestic violence and were excluded from the outcome analyses. No formal comparison was made between respondents and non-respondents, as the sociodemographic and psychosocial variables of interest are mostly in the same section of the instrument that contains the question about violence, resulting in incomplete data for non-respondents.

Variables and data sources

The variables analyzed in this study are part of the "PREGNANT WOMAN" section of questionnaire variables collected in the Curitiba Maternal and Child Health Cohort. The questionnaire was administered in a private setting at prenatal care services, conducted by interviewers trained in ethical and confidential practices, in order to minimize underreporting and ensure the internal validity of the information collected.

The dependent variable was "domestic violence", derived from the question: "Have you ever experienced violence at home?" (physical, psychological, sexual violence, abandonment, or no). Participants could select more than one option, making it possible to report different types of violence simultaneously.

For the analyses, the variable was dichotomized (yes; no), considering any type of violence reported. This decision was made based on the low frequency of the specific categories, which would make separate analyses with adequate statistical power impossible. However, the specific types of violence are presented descriptively (Figure 1).

The "current household arrangement" variable was obtained by asking the question: "Who do you currently live with?" The answers were grouped into the following categories: lives alone; with husband or partner; with husband/partner and children; with husband/partner and other people; or with other people. This latter category included pregnant women who reported living with family members (parents, siblings, uncles/aunts, grandparents) or friends, without a partner.

Gestational risk was classified, according to the Curitiba Mother Network protocol, into three categories: usual, intermediate and high. Clinical, obstetric and socioeconomic factors recorded during prenatal visits were considered. The independent variables used in this study are described in Table 1.

**Figure 1:**
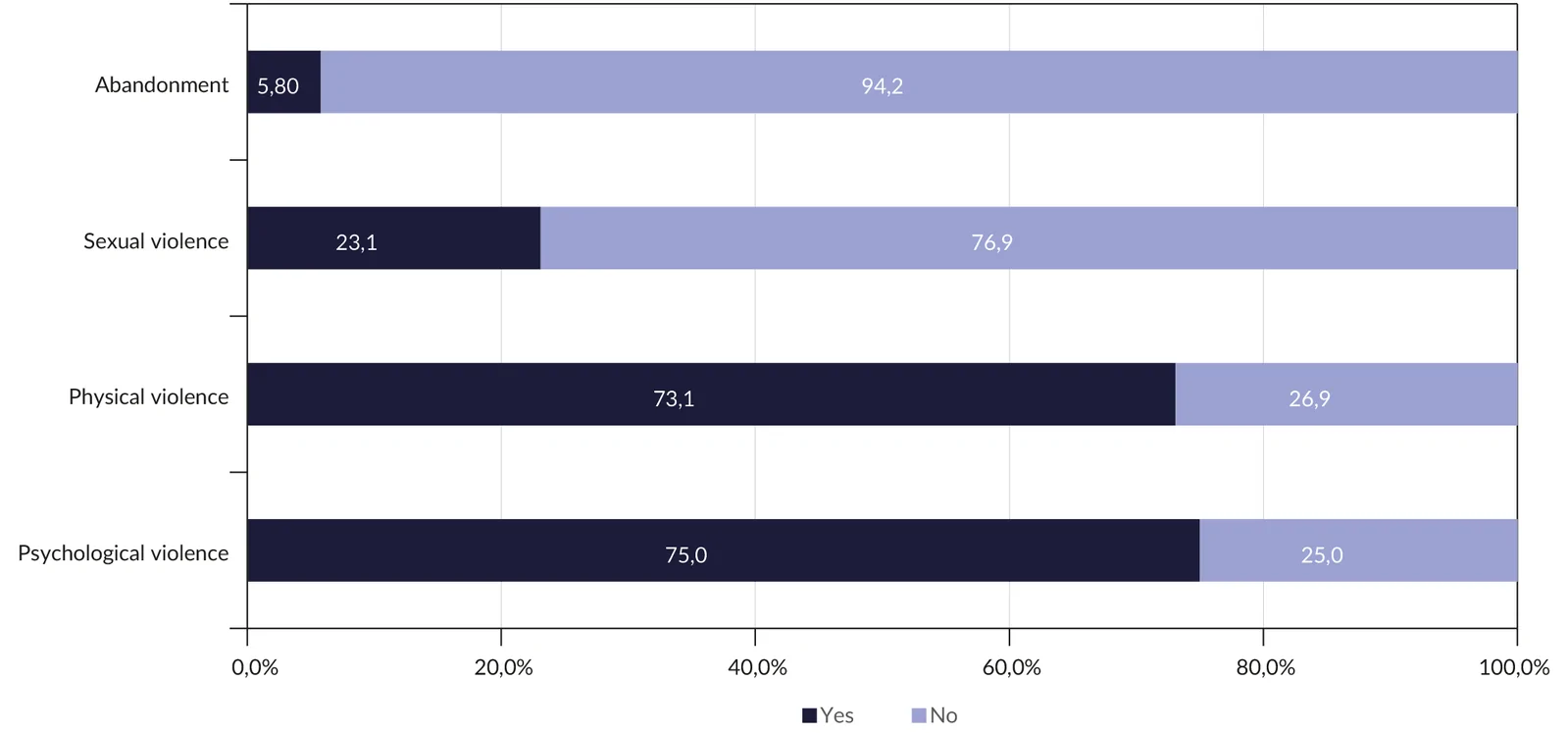
Distribution of type of reported domestic violence among pregnant women using public health services. Curitiba, 2018-2020 (n=52)

Statistical methods

Sample characterization was performed by calculating absolute and relative frequencies. The chi-square test was applied for the bivariate analysis, with Bonferroni correction to reduce the probability of type I errors resulting from multiple comparisons between independent variables and the outcome. This correction was performed automatically using IBM SPSS Statistics software and was used only as an exploratory screening criterion to select variables with p-value<0.20 for inclusion in the multivariate analysis. The multivariate analysis was conducted using Poisson regression with robust variance, employing the backward elimination technique to select variables for the final model.

Before modeling, we assessed presence of multicollinearity between the independent variables using the variance inflation factor, adopting a cutoff point of <10. Bivariate interactions between theoretically plausible variables (e.g., mother's feelings versus gestational risk and marital status versus household) were tested, but without statistical significance, which is why they were not retained in the final model.

Associations with p-value<0.05 and a 95% confidence interval were considered to be statistically significant. Analyses were performed using IBM SPSS Statistics software, version 25.0.

The bivariate analyses are shown in Tables 2, 3, and 4) and the results of the final adjusted model, obtained by Poisson regression with robust variance, is shown in Table 5. The option to present only the adjusted prevalence ratios aims to avoid redundancy and highlight independent associations, after simultaneously controlling for the other variables included in the model.

This manuscript was prepared in accordance with the recommendations of the Strengthening the Reporting of Observational Studies in Epidemiology (STROBE) statement ^14^.

**Table 1:** Summary of the variables and their respective categories, organized by domain, in relation to pregnant women using public health services. Curitiba, 2018-2020 (n=194)

Variables	Variable	Question	Categories
Maternal demographic	Age	Age (years)	16-19; 20-34; ≥35
	Race/skin color	What is your race/skin color?	White; Black; other
	Marital status	What is your marital status?	Single/separated; married/lives with partner
	Current household arrangement	Who do you currently live with?	Alone; husband/partner; husband/partner and children; husband/partner and other people; with other people
Maternal socioeconomic	Schooling	Up to which grade did you attend school?	Incomplete elementary or below; complete elementary or incomplete high school; complete high school; higher education - incomplete or above
	Work situation	Do you work?	No; yes
	Household income	What is the total approximate amount of your household income?	Up to 1 minimum wage; 1-3 minimum wages; 3-5 minimum wages; above 5 minimum wages
Service use	Emergency/Urgent care	Did you need emergency/urgent care during your pregnancy?	No; yes
	Psychological care	Did you receive care from a psychologist during your pregnancy?	No; yes
Gestational health	Gestational risk	Gestational risk?	High; intermediate; usual
Paternal sociodemographic characteristics	Father's age	How old is the father of your baby? (Years)	16-19; 20-34; ≥35
	Father's schooling	Up to which grade did he attend school?	Incomplete elementary or below; complete elementary; complete high school; higher education - incomplete or above
	Father's race/skin color	What is the race/skin color of the father of your baby?	White; Black; other
	Father's work situation	Does he work?	No; yes
Mental health	Mother's emotional reaction when discovering pregnancy	What was your reaction when you discovered you were pregnant?	Negative; neutral; positive
	Maternal feelings	Which of these face best shows how you are feeling, what you think about your life as a whole today?	Sad or very sad; neither sad nor happy; happy or very happy
	Father's reaction when discovering pregnancy	What was the baby's father's reaction when he discovered you were pregnant?	Negative; neutral; positive
	Father attends prenatal with mother	Has your partner attended your prenatal care sessions?	No; yes
	Family coexistence	Do you live with/have contact with your family (father, mother, grandparents, aunts and uncles)?	No; yes
	Excessive feeling of fear	Have you ever felt excessive fear at some time in your life?	No; yes

## Results 

In all, 194 participants answered the question about domestic violence and were included in the analyses. Of these, 27.5% (p-value<0.001) reported having suffered some type of violence. Among the types mentioned, 73.1% reported physical violence and 75.0% psychological violence, while 23.1% mentioned sexual violence and 5.8% reported situations of abandonment. Participants could indicate more than one category of violence, so the proportions were not mutually exclusive.

Most pregnant women, both those who reported experiencing domestic violence and those who did not, were between 20 and 34 years old (69.8%; 70.0%) and self-identified as White (62.2%; 61.5%) (Table 2). There was a considerable difference between single or separated women who did not experience domestic violence (23.7%) and those who did experience it (43.3%) (Table 2).

Regarding the psychological aspects of the pregnant women, a higher frequency of feelings of sadness (15.0%) and excessive fear (57.7%) was observed among women who reported having suffered domestic violence (Table 3). Regarding gestational risk, the prevalence of violence among women at intermediate risk is noteworthy. In the group that did not suffer violence, 10.0% were at intermediate risk - this figure rose to 25.0% among those who did suffer violence (Table 3).

There was greater prevalence of violence among women whose baby's father did not work (23.5%) and whose baby's father did not attend prenatal appointments (35.2%), compared to women who did not experience violence (Table 4).

After the analysis had been adjusted, the variables associated with domestic violence were: mother's feelings, people with whom she lives, and gestational risk. Prevalence of violence was higher among women who reported feeling sad (PR 2.83; 95%CI 1.46; 5.48; p-value 0.002). Living with a husband (PR 0.51; 95%CI 0.29; 0.92; p-value 0.025) and living with a husband/partner and children (PR 0.34; 95%CI 0.16; 0.74; p-value 0.007) were protective factors. Prevalence of violence was also higher among pregnant women at intermediate gestational risk (PR 2.73; 95%CI 1.54; 4.84; p-value 0.010) (Table 5).

**Table 2:** Association between domestic violence and sociodemographic characteristics of pregnant women using public health services. Curitiba, 2018-2020 (n=194)

Variables	No	Yes	p-value
	n (%)	n (%)	
Age group (years)			0.643
16-19	16 (11.4)	4 (7.5)	
20-34	98 (70.0)	37 (69.8)	
≥35	26 (18.6)	12 (22.6)	
Race/skin color			0.906
White	85 (61.5)	33 (62.2)	
Black	16 (11.5)	5 (9.4)	
Other	37 (26.8)	15 (28.3)	
Marital status			0.007
Single or separated	33 (23.7)	23 (43.3)	
Married or living with a partner	106 (76.2)	30 (56.6)	
Current household arrangement			0.007
Lives alone	0	2 (3.7)	
Husband/partner	54 (38.8)	19 (35.8)	
Husband/partner and children	36 (25.8)	8 (15.0)	
Husband/partner and other people	25 (17.9)	4 (7.5)	
Lives with other people	24 (17.2)	20 (37.7)	
Work situation			0.632
No	61 (44.2)	25 (48.0)	
Yes	77 (55.7)	27 (51.9)	
Household income (minimum wages)			0.163
Up to 1	19 (15.4)	8 (17.7)	
1-3	37 (30.0)	21 (46.6)	
3-5	25 (20.3)	6 (13.3)	
>5	42 (34.1)	10 (22.2)	

Note: Numbers below 194 indicate lack of answers to the corresponding variable.

**Table 3:** Association between domestic violence and psychological and gestational health characteristics of pregnant women using public health services. Curitiba, 2018-2020 (n=194)

Variables	No	Yes	p-valor
	n (%)	n (%)	
Had psychological care			0.121
No	132 (94.9)	47 (88.6)	
Yes	7 (5.0)	6 (11.3)	
Mother's feelings			0.002
Sad or very sad	5 (3.5)	8 (15.0)	
Neither sad nor happy	28 (19.8)	16 (30.1)	
Happy or very happy	108 (76.5)	29 (54.7)	
Reaction when discovering pregnancy			0.457
Negative	59 (43.0)	23 (45.0)	
Neutral	9 (6.5)	1 (1.9)	
Positive	69 (50.3)	27 (52.9)	
Excessive feeling of fear			0.001
No	96 (68.0)	22 (42.3)	
Yes	45 (31.9)	30 (57.7)	
Family coexistence			0.723
No	11 (7.8)	5 (9.4)	
Yes	129 (92.1)	48 (90.5)	
Gestational risk			0.020
High	25 (21.4)	13 (27.1)	
Intermediate	12 (10.3)	12 (25.0)	
Usual	80 (68.4)	23 (47.9)	

a Numbers below 194 indicate lack of answers to the corresponding variable.

**Table 4:** Association between domestic violence and paternal sociodemographic characteristics (pregnant women using public health services). Curitiba, 2018-2020 (n=194)

Variables	No	Yes	p-valor
	n (%)	n (%)	
Father's age group (years)			0.660
16-19	8 (5.8)	3 (6.0)	
20-34	89 (64.9)	29 (58.0)	
≥ 35	40 (29.1)	18 (36.0)	
Father's work situation			0.003
No	11 (8.8)	12 (23.5)	
Yes	128 (92.0)	39 (76.5)	
Father attending prenatal care sessions			0.020
No	27 (19.1)	18 (35.2)	
Yes	114 (80.8)	33 (64.7)	
Father's schooling			0.234
Up to complete elementary or incomplete high school	9 (6.7)	6 (12.2)	
Complete high school	80 (59.7)	23 (46.9)	
Complete higher education	45 (33.5)	20 (40.8)	
Father's reaction when discovering pregnancy			0.308
Negative	26 (19.1)	13 (25.0)	
Neutral	7 (5.1)	5 (9.6)	
Positive	103 (75.7)	34 (65.3)	

a Numbers below 194 indicate lack of answers to the corresponding variable.

**Table 5:** Prevalence ratios (PR) and 95% confidence intervals (95%CI) for domestic violence, according to psychological, sociodemographic and health characteristics of pregnant women using public health services. Curitiba, 2018-2020 (n=194)

Variables	p-value	PR (95%CI)
Mother's feelings		
Sad or very sad	0.002	2.83 (1.46; 5.48)
Happy or very happy	0.222	0.69 (0.38; 1.26)
Neither sad nor happy	-	1
Current household arrangement		
Husband/partner	0.025	0.51 (0.29; 0.92)
Husband/partner and children	0.007	0.34 (0.16; 0.74)
Husband/partner and other people	0.050	0.26 (0.10; 1.66)
With other people	-	1
Gestational risk		
High	0.798	1.09 (0.54; 2.25)
Intermediate	0.010	2.74 (1.54; 4.84)
Usual	-	1

## Discussion 

This study identified high incidence of domestic violence among pregnant women cared for in the Brazilian Unified Health System in Curitiba, especially psychological and physical violence. We found that emotional factors, household conditions and gestational risk were associated with the presence of violence, pointing to the multifactorial and relational nature of this phenomenon ^15^. Among women receiving follow-up in primary care, the prevalence rate of violence against them was found to be 37%, 21% of whom were pregnant ^15^. Similar prevalence rates were found in the literature, with estimates of 37% throughout life and 35% during pregnancy ^16^. These percentages ​​are worrying, considering the impacts they can have on women's health in general and specifically on pregnant women's health. Violence, in addition to physical harm, can affect the mental health of the victim, causing depression and even suicide attempts ^8^^,^^15^. Specifically during pregnancy, in addition to affecting the pregnant mother, violence can cause negative outcomes at birth for the baby, such as prematurity, low birth weight and spontaneous abortion ^8^^,^^15^^,^^17^^,^^18^.

Among the types of violence reported, the most frequent were psychological violence, followed by physical violence. These findings corroborate what is reported in the literature ^15^^,^^16^^,^^19^. Although physical marks are more evident, psychological violence produces intense existential suffering. This type of violence is silent and does not leave visible signs like physical violence, but the impacts are significant both during pregnancy and the postpartum period ^17^.

Regarding psychological violence, the feeling of sadness reported by some of the pregnant women may reflect emotional vulnerability associated with experiencing violence, even though the study in question did not directly assess self-esteem ^20^. This pattern was also observed in our study, since women who reported having suffered violence presented feelings of sadness more frequently. In the model adopted by this study, the reference category was "neither sad nor happy", an option chosen because it represents an intermediate position and is more frequent among the participants, which favored more stable comparisons between the extremes of feeling. Even so, the interpretation of the results indicates that prevalence of violence was higher among pregnant women who stated they were sad or very sad compared to those who felt happy or very happy (61% versus 21%), which highlighted the impact of emotional conditions on the occurrence of the outcome.

Analyzing the situation of women who lived with other people rather than partners, violence was seen to occur even when the household was not shared with a partner (38%), indicating that violence can persist regardless of cohabitation. This finding suggests the complexity of the dynamics of violence, which can involve different interpersonal relationships throughout a woman's life ^21^^,^^22^.

Regarding intermediate gestational risk being present in most women who, in this study, report suffering abuse, it should be considered that classification of this risk specifically defines not only the woman's biological health, as in the case of high risk. It also considers issues related to individual socioeconomic characteristics, which may be an indicator of risk of domestic violence. Pregnant women classified in this risk category may not specifically present an illness. However, racial, ethnic, educational, reproductive history and age group factors also characterize higher risk of illness during pregnancy, childbirth and the postpartum period ^23^^,^^24^. We propose that these factors be further studied in the future in order to elucidate this relationship.

Considering the high prevalence of psychological violence (75%) and the low frequency of psychological care among women who suffered violence (11%) found in this study, there is a need for greater access to psychological care within the Brazilian Unified Health System in order to minimize the impacts that domestic violence can cause. An interdisciplinary approach to treatment is necessary, right from identifying the problem and providing guidance through to addressing its causes. This makes addressing it a significant challenge for healthcare professionals, as it is a complex, sensitive phenomenon that is difficult to approach within the context of health services. The literature indicates that failure of healthcare professionals to identify violence can contribute to the perpetuation and maintenance of the problem's invisibility, which can compromise the effectiveness of care and attention actions for women's health ^4^^,^^25^^,^^26^.

This study had some limitations. The dependent variable was obtained through self-reporting, which may be subject to information bias, especially underreporting, considering the sensitivity of the topic, in addition to the possible apprehension of participants in reporting their experiences. Another point to consider was the dichotomization of the "domestic violence" variable, which, although necessary to enable analysis, may have limited identification of specific associations according to the type of violence (physical, psychological or sexual). Despite this, the approach adopted allowed for the description of the overall magnitude of the problem and the main factors correlated with its occurrence.

The outcome non-response rate was 20%. Although desirable, it was not possible to compare the profile of participants who did not respond with that of those who did, due to the unavailability/fragmentation of sociodemographic and psychosocial variables in the non-respondent group (same section of the questionnaire). This fact may limit representativeness and introduce information bias, and should be considered in the interpretation of the results. Future data collection could mitigate this problem by ensuring that minimum variables are recorded before administering sensitive sections of the questionnaire.

The study sample was obtained by convenience, including pregnant women linked to the Curitiba Mother Network. This selection limits the external validity of the results, which should be interpreted with caution regarding generalization to other populations. Although these factors may limit generalization of the findings, the data analyzed offer important elements for understanding the occurrence of domestic violence among pregnant women attending the public health network.

Three out of every ten pregnant women who participated in the research suffered or had suffered domestic violence at some point in their lives. The main types were physical and psychological violence. It was shown that women with intermediate gestational risk, who felt sad, and who did not live with a partner suffered more violence. These results highlight the importance of the topic as a public health problem and reinforce the need for intersectoral actions aimed at early identification and support for pregnant women in situations of violence.

In clinical practice, we recommend that prenatal services adopt systematic screening and reception routines, strengthening the linkage between service users and health professionals and ensuring referral flows. In the field of public policy, the findings can support strategies for women's health care and monitoring domestic violence.

## Data Availability

The data are available upon request by peer reviewers.
