# Supplementary material for: Prevalence of domestic violence and associated factors among pregnant women using the Brazilian Unified Health System: a cross-sectional study, Curitiba, 2018-2020
Source: Epidemiol Serv Saude. 2026 Jun 7;35:e20250920. doi: 10.1590/S2237-96222026v35e20250920.en (PMC13686310; doi:10.1590/S2237-96222026v35e20250920.en)
Supplement: Translation

# Translation: Prevalência da violência doméstica e fatores associados em gestantes usuárias do Sistema Único de Saúde: estudo transversal, Curitiba, 2018-2020

**DOI:** 10.1590/S2237-96222026v35e20250920.pt

**Authors:** Julia Valoski, Ádelin Olívia Lopes Joly Rodrigues, Isabela Cristina Santos Freire de Paula, Renata Iani Wernek, Juliana Schaia Rocha Orsi

**Affiliations:** ¹ Pontifícia Universidade Católica do Paraná, Curitiba, PR, Brasil Pontifícia Universidade Católica do Paraná Curitiba PR Brasil

**Keywords:** Violência Doméstica, Gestantes, Violência de Gênero, Serviços de Saúde, Estudos Transversais.

## Abstract

**Objetivo:**

Analisar a prevalência e os fatores associados à violência doméstica em gestantes atendidas na rede pública de Curitiba, a partir de dados do projeto Coorte de Saúde Materno-Infantil de Curitiba.

**Métodos:**

Estudo observacional transversal, conduzido com gestantes usuárias do Sistema Único de Saúde de Curitiba entre 2018 e 2020. Foram analisadas variáveis demográficas, socioeconômicas, emocionais e obstétricas. A variável dependente foi a experiência de violência doméstica (física, psicológica ou sexual). Calcularam-se frequências absolutas e relativas e aplicou-se o teste do qui-quadrado com correção de Bonferroni para seleção de variáveis com p-valor<0,20. As associações independentes foram estimadas por regressão de Poisson com variância robusta, apresentando razões de prevalência (RP) e intervalos de confiança de 95% (IC95%).

**Resultados:**

Foram analisadas 194 gestantes. A prevalência de violência doméstica foi de 27,5%, sendo 73,1% física e 75,0% psicológica. A violência esteve associada ao sentimento de tristeza (RP 2,83; IC95% 1,46; 5,48; p-valor 0,002), ao arranjo domiciliar (gestantes que moravam com o companheiro apresentaram menor prevalência de violência: RP 0,51; IC95% 0,29; 0,92; p-valor 0,025) e ao risco gestacional intermediário (RP 2,73; IC95% 1,54; 4,84; p-valor 0,011).

**Conclusão:**

A violência doméstica foi frequente entre gestantes atendidas na rede pública de Curitiba e associou-se à vulnerabilidade emocional, à moradia sem companheiro e ao risco gestacional intermediário. Esses achados reforçam a necessidade de ações intersetoriais e de apoio psicológico e social durante o pré-natal para identificação e manejo da violência.

Aspectos éticos

Esta pesquisa respeitou os princípios éticos, obtendo os seguintes dados de aprovação:

Comitê de ética em pesquisa: Pontifícia Universidade Católica do Paraná. Número do parecer: 6.498.474. Data de aprovação: 9/11/2023. Certificado de apresentação de apreciação ética: 90052118.9.3001.0101. Comitê de ética em pesquisa: Secretaria Municipal de Saúde de Curitiba. Número do parecer: 6.588.994. Data de aprovação: 18/12/2023. Certificado de apresentação de apreciação ética: 90052118.9.0000.0020. Registro do consentimento livre e esclarecido: obtido de todas as participantes antes da coleta.

## Introdução 

A violência contra a mulher é uma questão histórico-cultural presente de forma endêmica em todo o mundo e pode se manifestar de diversas maneiras como ofensa verbal, psicológica, agressão física, tortura e morte ^1^^,^^2^. A violência ocorre devido às iniquidades de gênero. Estas são construídas não apenas com base nas diferenças biológicas, mas sobretudo pelo papel socialmente atribuído ao homem, em uma civilização moldada por um ideal masculinizado e patriarcal. Nela, o homem é visto como figura central enquanto a mulher desempenha um papel secundário ^2^. Com diversas mudanças ao longo dos tempos, a exemplo do desenvolvimento tecnológico e sociocultural, além da evolução humana em si, foram travadas muitas lutas, movimentos e discussões a respeito do patriarcado ^1^^-^^3^.

O crescimento das notificações de violência doméstica, sexual e de outras formas de violência contra mulheres evidencia a persistência desse problema como um importante desafio para a saúde pública. Em 2023, foram registradas 302.856 notificações desse tipo de violência, representando um aumento expressivo em relação aos 216.024 casos registrados em 2022. Além de um problema sociocultural, a violência doméstica também se enquadra em questão de saúde pública ^4^^,^^5^. 

No Brasil, apesar dos avanços alcançados a partir da promulgação da Lei nº 11.340 (Lei Maria da Penha) em 2006, como uma resposta do Estado em relação a essas lutas ^6^, a violência contra a mulher segue enraizada em padrões socioculturais que limitam mudanças. Ademais, a efetividade das respostas institucionais não depende apenas da penalização dos culpados, mas também da ampliação de estratégias de prevenção, identificação precoce e acolhimento ^7^^,^^8^.

Quando se trata da mulher em período gestacional, a violência assume relevância ainda maior, uma vez que contribui para o aumento da taxa de morbidade e mortalidade materna. Nesse sentido, a incorporação sistemática da identificação da violência contra gestantes nas ações de saúde pode contribuir para a visibilidade desse e possibilitar intervenções mais eficazes para reduzir índices ^9^^,^^10^. 

Os serviços básicos de saúde trabalham na detecção, prevenção e ação contra a violência. Eles são considerados a porta de entrada para se abordar esse assunto e estabelecer vínculo com as mulheres durante a assistência pré-natal, identificando a situação e encorajando a notificação ^7^^,^^11^. No Brasil, a escassez de estudos sobre a ocorrência e os tipos de violência em mulheres nesse período pode estar relacionada à dificuldade que as mulheres encontram em relatar a violência, tanto em contextos institucionais quanto em pesquisas, o que pode contribuir para a ocorrência de uma subnotificação desse fenômeno ^7^^,^^12^.

Este estudo teve como objetivo analisar a prevalência de violência doméstica e fatores associados em gestantes atendidas no Sistema Único de Saúde de Curitiba.

## Métodos 

Delineamento do estudo

Trata-se de um estudo observacional transversal, realizado com base em um recorte da Coorte de Saúde Materno-Infantil de Curitiba. Foram utilizadas apenas as informações coletadas durante o período gestacional, o que configurou uma análise de corte transversal, selecionada para estimar a prevalência da violência doméstica e identificar fatores associados à gestação ^13^.

Contexto

A Coorte de Saúde Materno-Infantil de Curitiba é um estudo observacional e longitudinal de coorte mista (histórica e concorrente) focado em gestantes e seus filhos, todos vinculados à rede pública de saúde de Curitiba, Paraná, Brasil. A metodologia detalhada da coorte, incluindo o instrumento de coleta e seu processo de validação, encontra-se descrita em seu protocolo metodológico ^11^. A coorte concorrente envolve o recrutamento de gestantes, em qualquer estágio da gravidez, com 16 anos ou mais, residentes em Curitiba. 

O objetivo da Coorte de Saúde Materno-Infantil de Curitiba é explorar diversos aspectos da saúde de gestantes e seus filhos durante os primeiros 1 mil dias de vida. Isso contribui para a compreensão de fatores que impactam o desenvolvimento infantil e o bem-estar materno no contexto da rede Mãe Curitibana.

Participantes 

Trata-se de amostra de conveniência, composta de todas as gestantes participantes do estudo da Coorte de Saúde Materno-Infantil de Curitiba entre agosto de 2018 e maio de 2020 (n=244) que responderam à pergunta sobre violência doméstica (n=194). Não foi realizado cálculo amostral, uma vez que este estudo constitui uma análise secundária de dados disponíveis. 

As participantes que aceitaram integrar o estudo assinaram o registro de consentimento livre e esclarecido. A coleta foi realizada presencialmente por equipe previamente treinada, por meio de um questionário estruturado em blocos de variáveis. O protocolo completo pode ser consultado para mais detalhes sobre o estudo ^13^. 

Do total de 244 gestantes elegíveis, 50 (20,49%) não responderam à pergunta sobre violência doméstica e foram excluídas das análises relativas ao desfecho. Não foi realizada comparação formal entre respondentes e não respondentes, pois as variáveis sociodemográficas e psicossociais de interesse estão majoritariamente no mesmo bloco do instrumento que contém a pergunta sobre violência, resultando em dados incompletos para as não respondentes. 

Variáveis e fontes de dados

As variáveis analisadas neste estudo fazem parte do bloco "GESTANTE" coletado na Coorte de Saúde Materno-Infantil de Curitiba. O questionário foi aplicado em ambiente reservado nos serviços de pré-natal, conduzidos por entrevistadoras treinadas para abordagem ética e sigilosa, a fim de minimizar a subnotificação e garantir a validade interna das informações coletadas. 

A variável dependente foi "violência doméstica", obtida pela pergunta: "Você já sofreu violência dentro de casa?" (violência física, psicológica, sexual, abandono ou não). As participantes puderam assinalar mais de uma alternativa, sendo possível o relato de diferentes tipos de violência concomitantemente. 

Para as análises, a variável foi dicotomizada (sim; não), considerando qualquer tipo de violência relatada. Essa decisão foi tomada com base na baixa frequência das categorias específicas, o que impossibilitaria análises separadas com poder estatístico adequado. Entretanto, os tipos específicos de violência foram apresentados de forma descritiva (Figura 1). 

A variável "moradia atual" foi obtida por meio da pergunta: "Com quem você mora atualmente?". As respostas foram agrupadas nas seguintes categorias: mora sozinha; com marido ou companheiro; com marido/companheiro e filhos; com marido/companheiro e outros; e com outras pessoas. Esta última categoria incluiu gestantes que relataram residir com familiares (pais, irmãos, tios, avós) ou amigos, sem a presença de parceiro. 

O risco gestacional foi classificado, conforme o protocolo da rede Mãe Curitibana, em três categorias: habitual, intermediário e alto. Consideraram-se fatores clínicos, obstétricos e socioeconômicos registrados nas consultas de pré-natal. As variáveis independentes utilizadas neste estudo estão descritas na Tabela 1.

**Figura 1:** Distribuição do tipo de violência doméstica relatada por gestantes usuárias do serviço público. Curitiba, 2018-2020 (n=52)

Métodos estatísticos

A caracterização da amostra foi realizada por meio do cálculo de frequências absolutas e relativas. Para a análise bivariada, aplicou-se o teste do qui-quadrado com correção de Bonferroni, com o objetivo de reduzir a probabilidade de erro tipo I decorrente das múltiplas comparações entre as variáveis independentes e o desfecho. A correção foi realizada automaticamente no software IBM SPSS Statistics, sendo utilizada apenas como critério exploratório de triagem para selecionar variáveis com p-valor<0,20 a serem incluídas na análise multivariada. Esta foi conduzida por regressão de Poisson com variância robusta, utilizando a técnica de eliminação *Backward* para seleção das variáveis no modelo final. 

Antes da modelagem, avaliou-se a presença de multicolinearidade entre as variáveis independentes por meio do fator de inflação da variância, adotando-se o ponto de corte<10. Foram testadas interações bivariadas entre variáveis teoricamente plausíveis (por exemplo, sentimento da mãe×risco gestacional e estado civil×moradia), sem significância estatística, motivo pelo qual não foram mantidas no modelo final. 

Foram consideradas estatisticamente significativas as associações com p-valor<0,05 e intervalo de confiança de 95%. As análises foram realizadas no software IBM SPSS Statistics, versão 25.0. 

Foram apresentadas as análises bivariadas (Tabelas 2, 3 e 4) e os resultados do modelo ajustado final, obtido por regressão de Poisson com variância robusta (Tabela 5). A opção de exibir exclusivamente as razões de prevalência ajustadas visa evitar redundância e destacar as associações independentes, após o controle simultâneo das demais variáveis incluídas no modelo.

Este manuscrito foi elaborado conforme as recomendações do checklist Strengthening the Reporting of Observational Studies in Epidemiology para estudos observacionais ^14^.

**Tabela 1:** Resumo das variáveis e suas respectivas categorias, organizado por domínios, com relação às gestantes usuárias do serviço público. Curitiba, 2018-2020 (n=194)

Variáveis	Variável	Pergunta	Categorias
Demográficos	Idade	Idade (anos)	16-19; 20-34; ≥35
	Raça/cor da pele	Qual é a sua raça/cor da pele?	Branca; preta; outras
	Estado civil	Qual o seu estado conjugal?	Solteira/separada; casada/vive com companheiro
	Arranjo domiciliar atual	Com quem você mora atualmente?	Sozinha; marido companheiro; marido companheiro e filhos; marido/companheiro e outros; com outras pessoas
Socioeconômicos da mãe	Escolaridade	Até qual série você estudou?	Fundamental incompleto ou menos; fundamental completo ou médio incompleto; médio completo; superior incompleto ou mais
	Situação de trabalho	Você trabalha?	Não; sim
	Renda domiciliar	Qual é o valor total aproximado da renda do domicílio?	Até 1 salário mínimo; 1-3 salários mínimos; 3-5 salários mínimos; acima de 5 salários mínimos
Uso de serviços	Atendimento de emergência/urgência	Você já precisou de atendimento de emergência/urgência durante a gestação?	Não; sim
	psicológico	Você foi atendida por um psicólogo durante a gestação?:	Não; sim
Saúde gestacional	Risco gestacional	Risco gestacional?	Alto; intermediário; habitual
Características sociodemográficas do pai	Idade do pai	Qual a idade do pai do seu bebê? (anos)	16-19; 20-34; ≥35
	Escolaridade do pai	Até qual série ele estudou?	Fundamental incompleto ou menos; fundamental completo; ensino médio; superior incompleto ou mais
	Raça/cor da pele do pai	Qual a raça/cor da pele do pai do seu bebê?	Branca; preta; outras
	Situação de trabalho do pai	Ele trabalha?	Não; sim
Saúde mental	Reação emocional ao descobrir gravidez	Qual sua reação quando soube da sua gravidez?	Negativa; neutra; positiva
	Sentimento da mãe	Qual destas faces mostra melhor como você se sente, pensando em sua vida como um todo hoje?	Triste ou muito triste; nem triste nem feliz; feliz ou muito feliz
	Reação paterna ao descobrir gravidez	Qual a reação do pai da criança quando soube da sua gravidez?	Negativa; neutra; positiva
	Pai acompanha pré-natal	O seu(sua) parceiro(a) tem acompanhado as suas consultas no pré-natal?	Não; sim
	Convívio familiar	Você convive/tem contato com sua família (pai, mãe, avós, tios)?	Não; sim
	Sentimento de medo excessivo	Você já teve medo excessivo em algum período da sua vida?	Não; sim

## Resultados 

Ao total, 194 participantes responderam à pergunta sobre violência doméstica e foram incluídas nas análises. Destas, 27,5% (p-valor<0,001) relataram ter sofrido algum tipo de violência. Entre os tipos referidos, 73,1% relataram violência física e 75,0% violência psicológica, enquanto 23,1% mencionaram violência sexual e 5,8% relataram situações de abandono. As participantes puderam assinalar mais de uma categoria de violência, de modo que as proporções não foram mutuamente excludentes.

A maioria das gestantes, tanto as que relataram ter sofrido violência doméstica quanto as que negaram, tinham idade entre 20 e 34 anos (69,8%; 70,0%) e se autodeclarava branca (62,2%; 61,5%) (Tabela 2). Houve diferença considerável entre mulheres solteiras ou separadas que não sofreram (23,7%) e que sofreram (43,3%) violência doméstica (Tabela 2).

Sobre os aspectos psicológicos da gestante, observou-se maior frequência de sentimento de tristeza (15,0%) e de medo excessivo(57,7%) entre as mulheres que relataram ter sofrido violência doméstica (Tabela 3). A respeito do risco gestacional, chama atenção a prevalência de violência entre as mulheres de risco intermediário. No grupo que não sofreu violência, 10,0% eram do risco intermediário - o valor subiu para 25,0% entre aquelas que sofreram (Tabela 3).

Houve maior prevalência de violência entre as mulheres cujo pai da criança não trabalhava (23,5%) e cujo pai não acompanhava o pré-natal (35,2%), comparando-se com as mulheres que não sofreram violência (Tabela 4).

Após a análise ajustada, as variáveis associadas à violência doméstica foram: sentimento da mãe, pessoas com quem reside e risco gestacional. Mulheres que relataram estar tristes apresentaram maior prevalência de violência (RP 2,83; IC95% 1,46; 5,48; p-valor 0,002). Morar com o marido (RP 0,51; IC95% 0,29; 0,92; p-valor 0,025) e morar com o companheiro e filhos (RP 0,34; IC95% 0,16; 0,74; p-valor 0,007) foram fatores protetivos. Gestantes de risco intermediário também apresentaram prevalência mais elevada de violência (RP 2,73; IC95% 1,54; 4,84; p-valor 0,010) (Tabela 5)

**Tabela 2:** Associação entre violência doméstica e características sociodemográficas das gestantes usuárias do serviço público. Curitiba, 2018-2020 (n=194)

Variáveis	Não	Sim	p-valor
	n (%)	n (%)	
Faixa etária (anos)			0,643
16-19	16 (11,4)	4 (7,5)	
20-34	98 (70,0)	37 (69,8)	
≥35	26 (18,6)	12 (22,6)	
Raça/cor da pele			0,906
Branca	85 (61,5)	33 (62,2)	
Preta	16 (11,5)	5 (9,4)	
Outras	37 (26,8)	15 (28,3)	
Estado civil			0,007
Solteira ou separada	33 (23,7)	23 (43,3)	
Casada ou vive com companheiro	106 (76,2)	30 (56,6)	
Arranjo domiciliar atual			0,007
Sozinha	0	2 (3,7)	
Marido ou companheiro	54 (38,8)	19 (35,8)	
Marido ou companheiro e filhos	36 (25,8)	8 (15,0)	
Marido ou companheiro e outros	25 (17,9)	4 (7,5)	
Com outras pessoas	24 (17,2)	20 (37,7)	
Situação de trabalho			0,632
Não	61 (44,2)	25 (48,0)	
Sim	77 (55,7)	27 (51,9)	
Renda domiciliar (salários mínimos)			0,163
Até 1	19 (15,4)	8 (17,7)	
1-3	37 (30,0)	21 (46,6)	
3-5	25 (20,3)	6 (13,3)	
>5	42 (34,1)	10 (22,2)	

Valores inferiores a 194 indicam ausência de resposta para a variável correspondente.

**Tabela 3:** Associação entre violência doméstica e características psicológicas e de saúde gestacional das gestantes usuárias do serviço público. Curitiba, 2018-2020 (n=194)

Variáveis	Não n (%)	Sim n (%)	p-valor
	n (%)	n (%)	
Realizou atendimento psicológico			0,121
Não	132 (94,9)	47 (88,6)	
Sim	7 (5,0)	6 (11,3)	
Sentimento da mãe			0,002
Triste ou muito triste	5 (3,5)	8 (15,0)	
Nem triste e nem feliz	28 (19,8)	16 (30,1)	
Feliz ou muito feliz	108 (76,5)	29 (54,7)	
Emoção ao descobrir gravidez			0,457
Negativa	59 (43,0)	23 (45,0)	
Neutra	9 (6,5)	1 (1,9)	
Positiva	69 (50,3)	27 (52,9)	
Sentimento de medo excessivo			0,001
Não	96 (68,0)	22 (42,3)	
Sim	45 (31,9)	30 (57,7)	
Convívio familiar			0,723
Não	11 (7,8)	5 (9,4)	
Sim	129 (92,1)	48 (90,5)	
Risco gestacional			0,020
Alto	25 (21,4)	13 (27,1)	
Intermediário	12 (10,3)	12 (25,0)	
Habitual	80 (68,4)	23 (47,9)	

a Valores inferiores a 194 indicam ausência de resposta para a variável correspondente.

**Tabela 4:** Associação entre violência doméstica e características sociodemográficas do pai (gestantes usuárias do serviço público). Curitiba, 2018-2020 (n=194)

Variáveis	Não n (%)	Sim n (%)	p-valor
Faixa etária do pai (anos)			0,660
16-19	8 (5,8)	3 (6,0)	
20-34	89 (64,9)	29 (58,0)	
≥ 35	40 (29,1)	18 (36,0)	
Situação de trabalho do pai			0,003
Não	11 (8,8)	12 (23,5)	
Sim	128 (92,0)	39 (76,5)	
Acompanhamento pré-natal pelo pai			0,020
Não	27 (19,1)	18 (35,2)	
Sim	114 (80,8)	33 (64,7)	
Escolaridade do pai			0,234
Até fundamental completo ou médio incompleto	9 (6,7)	6 (12,2)	
Médio completo	80 (59,7)	23 (46,9)	
Superior completo	45 (33,5)	20 (40,8)	
Reação paterna ao descobrir gravidez			0,308
Negativa	26 (19,1)	13 (25,0)	
Neutra	7 (5,1)	5 (9,6)	
Positiva	103 (75,7)	34 (65,3)	

a Valores inferiores a 194 indicam ausência de resposta para a variável correspondente.

**Tabela 5:** Razão de prevalências (RP) e intervalos de confiança de 95% (IC95%) da violência doméstica, segundo características psicológicas, sociodemográficas e de saúde das gestantes usuárias do serviço público. Curitiba, 2018-2020 (n=194)

Já sofreu violência em casa?		
Variáveis	p-valor	RP (IC95%)
Sentimentos da mãe		
Triste ou muito triste	0,002	2,83 (1,46; 5,48)
Feliz ou muito feliz	0,222	0,69 (0,38; 1,26)
Nem triste e nem feliz	-	1
Arranjo domiciliar atual		
Marido ou companheiro	0,025	0,51 (0,29; 0,92)
Marido ou companheiro e filhos	0,007	0,34 (0,16; 0,74)
Marido ou companheiro e outros	0,050	0,26 (0,10; 1,66)
Com outras pessoas	-	1
Risco gestacional		
Alto	0,798	1,09 (0,54; 2,25)
Intermediário	0,010	2,74 (1,54; 4,84)
Habitual	-	1

## Discussão 

Este estudo identificou elevada ocorrência de violência doméstica entre gestantes atendidas no Sistema Único de Saúde de Curitiba, especialmente nas formas psicológica e física. Observou-se que fatores emocionais, condições de moradia e risco gestacional estiveram associados à presença de violência, apontando para a natureza multifatorial e relacional desse fenômeno ^15^. Entre mulheres acompanhadas pela atenção primária, foi encontrada prevalência de 37% de violência contra a mulher, entre as quais 21% estavam grávidas ^15^. Prevalências semelhantes foram observadas na literatura, com estimativas de 37% ao longo da vida e 35% na gravidez ^16^. Esses valores são preocupantes, tendo em vista os impactos que eles podem causar na saúde da mulher e especificamente da gestante. Agressões, além dos danos físicos, podem afetar a saúde mental da pessoa agredida, causando depressão e até mesmo tentativa de suicídio ^8^^,^^15^. Especificamente durante a gestação, além de afetar a mulher, a agressão pode causar desfechos negativos no nascimento do bebê, como prematuridade, baixo peso ao nascer e aborto espontâneo ^8^^,^^15^^,^^17^^,^^18^.

Dos tipos de violência reportados, os mais frequentes foram a violência psicológica, seguida da física. Esses achados corroboram o reportado na literatura ^15^^,^^16^^,^^19^. Apesar de as marcas físicas serem mais evidentes, a violência psicológica produz um sofrimento existencial intenso. Esse tipo de violência é silencioso e não deixa sinais visíveis como a física, mas os impactos são significativos tanto na gravidez quanto no puerpério ^17^.

Ainda sobre a violência psicológica, o sentimento de tristeza relatado pelas gestantes pode refletir vulnerabilidade emocional associada à experiência de violência, ainda que este estudo não tenha avaliado diretamente a autoestima ^20^. Esse padrão também foi observado no presente estudo, uma vez que as mulheres que relataram ter sofrido violência apresentaram com mais frequência sentimentos de tristeza. No modelo adotado por este estudo, a categoria-referência foi "nem triste nem feliz", opção adotada por representar uma posição intermediária e de maior frequência entre as participantes, o que favoreceu comparações mais estáveis entre os extremos de sentimento. Ainda assim, a interpretação dos resultados indica que gestantes que se declararam tristes ou muito tristes apresentaram maior prevalência de violência em relação àquelas que se sentiam felizes ou muito felizes (61% vs. 21%), o que evidenciou o impacto das condições emocionais sobre a ocorrência do desfecho.

Analisando a situação de mulheres que moram com outras pessoas e não companheiros, observa-se a ocorrência de violência mesmo na ausência de moradia conjunta com o parceiro (38%), indicando que a violência pode persistir independente da coabitação. Esse achado sugere a complexidade das dinâmicas de violência, que podem envolver diferentes vínculos interpessoais ao longo da vida da mulher ^21^^,^^22^.

Em relação ao risco gestacional intermediário estar presente na maioria das mulheres que, neste estudo, afirmam sofrer agressão, deve-se levar em consideração que a classificação especificamente desse risco não define apenas a saúde biológica da mulher, como no alto risco. Ele também considera questões relacionadas às características socioeconômicas individuais, as quais podem ser um indicativo de risco à violência doméstica. Gestantes enquadradas nesse risco podem não apresentar especificamente uma doença. Porém, fatores raciais, étnicos, educacionais, história da vida reprodutiva e de faixa etária também caracterizam um risco maior para adoecer durante a gestação, o parto e o pós-parto ^23^^,^^24^. Propõe-se que esses fatores sejam mais estudados futuramente, a fim de elucidar essa relação.

Considerando a alta prevalência de violência psicológica (75%) e a baixa frequência de realização de atendimentos psicológicos entre as mulheres que sofreram violência (11%) encontrados neste estudo, nota-se a necessidade de maior acesso à atendimento com psicólogos no Sistema Único de Saúde, para minimizar os impactos que a violência doméstica pode causar. É necessário que haja uma base interdisciplinar para o tratamento, desde a identificação do problema, da orientação e por fim das causas. Isso torna seu enfrentamento um desafio relevante para os profissionais da saúde, uma vez que se trata de um fenômeno complexo, sensível e de difícil abordagem no contexto dos serviços. A literatura aponta que a não identificação da violência pelos profissionais de saúde pode contribuir para a perpetuação e manutenção da invisibilidade do problema, o que pode comprometer a efetividade das ações de cuidado e atenção à saúde da mulher ^4^^,^^25^^,^^26^.

Este estudo apresentou algumas limitações. A variável dependente foi obtida por meio de autorrelato, o que pode estar sujeito a viés de informação, especialmente subnotificação, considerando que o tema é sensível, além do possível receio das participantes em relatar situações vivenciadas. Outro ponto a ser considerado foi a dicotomização da variável "violência doméstica", que, embora necessária para viabilizar a análise, pode ter limitado a identificação de associações específicas segundo o tipo de violência (física, psicológica ou sexual). Apesar disso, a abordagem adotada permitiu descrever a magnitude global do problema e os principais fatores correlacionados à sua ocorrência. 

A taxa de não resposta ao desfecho foi de 20%. Embora desejável, não foi possível comparar o perfil das participantes que não responderam com o das que responderam, devido à indisponibilidade/fragmentação de variáveis sociodemográficas e psicossociais no grupo de não respondentes (mesmo bloco do instrumento). Esse fato pode limitar a representatividade e introduzir viés de informação, devendo ser considerado na interpretação dos resultados. Futuras coletas podem mitigar esse problema ao garantir o registro de variáveis mínimas antes de blocos sensíveis do questionário. 

A amostra do estudo foi obtida por conveniência, contemplando gestantes vinculadas à rede Mãe Curitibana. Esse recorte restringe a validade externa dos resultados, que devem ser interpretados com cautela quanto à generalização para outras populações. Embora esses fatores possam restringir a generalização dos achados, os dados analisados oferecem elementos importantes para compreender a ocorrência da violência doméstica entre gestantes atendidas na rede pública de saúde.

Três a cada 10 gestantes que participaram da pesquisa sofrem ou já sofreram violência doméstica em algum momento da vida. Os principais tipos foram a física e a psicológica. Demonstrou-se que mulheres com risco gestacional intermediário, que se sentiam tristes e que não moravam com o companheiro sofreram mais violência. Esses resultados evidenciam a importância do tema como problema de saúde pública e reforçam a necessidade de ações intersetoriais voltadas à identificação precoce e ao apoio às gestantes em situação de violência. 

Na prática assistencial, recomenda-se que os serviços de pré-natal adotem rotinas sistemáticas de triagem e acolhimento, fortalecendo o vínculo entre usuárias e profissionais e garantindo fluxos de encaminhamento. No campo das políticas públicas, os achados podem apoiar estratégias de atenção à mulher e o monitoramento desse agravo.

## Data Availability

Os dados estão disponíveis sob demanda dos pareceristas.
